# Corpus callosum integrity loss predicts cognitive impairment in Leukoaraiosis

**DOI:** 10.1002/acn3.51231

**Published:** 2020-10-29

**Authors:** Zhuonan Wang, Lijun Bai, Qi Liu, Shan Wang, Chuanzhu Sun, Ming Zhang, Yumei Zhang

**Affiliations:** ^1^ The Key Laboratory of Biomedical Information Engineering Ministry of Education Department of Biomedical Engineering School of Life Science and Technology Xi’an Jiaotong University Xi'an China; ^2^ Department of Medical Imaging The First Affiliated Hospital of Xi’an Jiaotong University Xi'an China; ^3^ Department of Neurology Beijing Tiantan Hospital Capital Medical University Beijing China; ^4^ Department of Rehabilitation Beijing Tiantan Hospital Capital Medical University Beijing China; ^5^ China National Clinical Research Center for Neurological Diseases Beijing China

## Abstract

**Objective:**

To investigate regional white matter fibers loss in Leukoaraiosis (LA) and its relationship with cognitive impairments.

**Methods:**

Fifty‐six participants with LA and 38 healthy controls underwent clinical evaluations and MR scans. Participants with LA were classified as cognitively normal (LA‐NC, *n* = 18), vascular cognitive impairment of none dementia (LA‐VCIND, *n* = 24), and vascular dementia (LA‐VaD, *n* = 14) by Mini‐Mental State Examination and Clinical Dementia Rating. Cognitive domains including visual‐spatial, naming, attention, language, abstraction, memory, and orientation were assessed. With the use of Tract‐based spatial statistics, mean fractional anisotropy (FA) of major white matter fiber tracts were compared between LA and controls and among LA groups with varying levels of cognitive impairments. Regression analyses were performed to evaluate relationships between FA values and cognitive performance.

**Results:**

Participants showed significant FA reduction in the corpus callosum (CC), bilateral corona radiata, anterior limb of the internal capsule, external capsule, posterior thalamic radiation, and superior longitudinal fasciculus compared to controls and across LA groups. The LA‐VaD group showed consistent damage in the body and genu of CC compared to the LA‐NC and LA‐VCIND groups. A positive correlation between visual‐spatial and FA reduction in right anterior corona radiates in LA‐VCIND and body of CC in LA‐ VaD.

**Interpretation:**

We found regional fiber loss in the CC across the cognitive spectrum in patients with LA and correlations between FA and visuospatial impairment in the anterior corona radiata in patients with LA‐VCIND and in the body of CC in patients with LA‐VaD.

## Introduction

Leukoaraiosis (LA), also known as the white matter hyperintensities (WMH) is characterized by cerebral white matter lesions due to insufficient blood supply. It impairs neuroplasticity through its adverse effects on white matter tract integrity.^1^, ^2^, ^3^, ^4^, ^5^ The severity and progression of LA are associated with cognitive impairments and increased risks for vascular dementia (VaD).^1^, ^3^, ^6^, ^7^, ^8^ LA precedes vascular cognitive impairment of none dementia (VCIND) and becomes highly prevalent in VaD over the disease course.^9^ Prior works have demonstrated that the LA Fazekas scale scores and regional distributions of WMH are associated with cognitive impairments;^10^, ^11^ however, the severity of the cognitive impairments cannot be effectively predicted. Moreover, the microstructural integrity loss of white matter fibers associated with the VaD degenerative process appears to change along with the degradation of cognition and has distinct region‐specific injury patterns as assessed by the fractional anisotropy (FA) changes in diffusion tensor imaging (DTI).^12^

The corpus callosum (CC) is a common target for investigating changes in VaD, given that it has a central role in the interhemispheric connectivity and coordination of cognitive function, as well as the highly topographic organization of its projections.^13^, ^14^ Specific changes in white matter integrity and structural changes in this region have been detected in an individual with dementia and LA.^15^, ^16^, ^17^ The progression of CC integrity loss is also found to be more rapid in individuals with LA who develop dementia.^16^, ^18^ Cross‐sectional studies have found that individuals with Alzheimer’s disease (AD) show lower FA in the genu and splenium of CC than healthy controls (HC), and lower FA in the genu than patients with mild cognitive impairment (MCI).^12^ Furthermore, the progression of neurodegenerative diseases is often characterized by the extent of white matter lesions in multiple sub‐regions of the CC. These relationships between the regional white matter microstructure deficits and functions of different cognitive domains, including visual‐spatial functions, are also commonly described in neurodegenerative diseases.^19^

The visual‐spatial deficits have been demonstrated to precede other cognitive impairments in VaD and are capable of distinguishing VCIND patients from cognitively normal (NC) participants.^4^, ^20^ However, the relationship between visual‐spatial impairments and callosum fiber loss in patients with LA and varying degrees of cognitive deterioration is still unclear. The present study aims to (1) investigate the between‐group differences in the entire skeleton of major white matter fiber tracts FA values in LA and HC participants. (2) Examine the relationship between white matter fiber loss and cognitive deficits by comparing the FA values across the LA groups with varying levels of cognitive impairments (i.e., LA‐NC, LA‐VCIND, and LA‐VaD). (3) Investigate whether the relationship between white matter fibers loss and visual‐spatial performance is impacted by the CC specific‐regional integrity of patients with LA who demonstrate varying degrees of cognitive impairments.

## Methods

### Participants

A total of 56 individuals with LA aged 50 to 85 years and 38 age‐, sex‐ and education matched healthy controls (HC; 50% females, mean age of 58.8 ± 10.8 years) were enrolled in this study. The referral and recruitment criteria of the participants were consistent with the Leukoaraiosis and Disability Study.^21^ In the current study, the inclusion criteria were as follows: (1) Presence of white matter hyperintensities of any degree detected through the MRI in accordance with a revised version of the scale of Fazekas.^21^ (2) None to mild impairments on the Instrumental Activities of Daily Living scale.^22^ (3) Availability of an informant. Participants were excluded if they presented with (1) severe illness likely leading to premature termination of participation (e.g., cardiac diseases, hepatic diseases or renal failure, cancer or other relevant systemic diseases), (2) severe unrelated neurological diseases, (3) leucoencephalopathy of non‐vascular origin (e.g., immunological‐demyelinating, metabolic, toxic, infectious or other origins), (4) severe psychiatric disorders, (5) current or history use of psychoactive drugs, or (6) contraindications to the MRI environment.^18^ All subjects gave written, informed consent in person approved by a local institutional review board. The research procedures were approved by the Ethical Committee of Beijing Tiantan Hospital, Capital Medical University, and were conducted in accordance with the Declaration of Helsinki.

The study protocol included MRI data acquisition, clinical imaging diagnosis of LA^23^, ^24^, and completion of Clinical Dementia Rating Scale (CDRS), Mini‐Mental State Examination (MMSE), and Montreal Cognitive Assessment (MoCA). All clinical scales were evaluated by two experienced neurologists. Based on the MRI images and the MMSE scores, the participants were divided into HC, LA‐NC, LA‐VCIND, and LA‐VaD groups (see Supplemental Materials for participant information in more detail).

### Neuropsychological Assessments

The MoCA is a brief neuropsychological assessment that evaluates seven cognitive domains, including visual‐spatial (assessed using a clock‐drawing task, a three‐dimensional cube copy, and an alternation task adapted from the Trail Making B task), naming (assessed using a three‐item confrontation naming task animals lion, camel, rhinoceros), attention (assessed using target detection using tapping, a serial subtraction task and digits span forward and backward), language (assessed using the repetition of two syntactically complex sentences and a phonemic fluency task), abstraction (assessed using a two‐item verbal abstraction task), memory (involves two learning trials of five nouns and delayed recall after approximately 5 minutes), and orientation (time and place are evaluated).^25^

### MRI acquisition and imaging analysis

The MRI acquisition sequences included T1‐weighted 3‐dimensional magnetization‐prepared rapid acquisition gradient‐echo, a single‐shot, spin echo‐based and diffusion‐weighted echo‐planar imaging sequence, T2‐weighted fast‐spin echo, and fluid‐attenuated inversion recovery images as described in Supplemental Materials. DTI data for the study were preprocessed using the FMRIB’s Diffusion Toolbox (FDT) in the FMRIB’s Software Library (FSL, http://www.fmrib.ox.ac.uk/fsl). The technical details of the preprocessing steps, quality control for DTI data, and tract‐based spatial statistics procedures can be found in Supplemental Materials.

### Statistical analysis

#### Clinical characteristics

MoCA subscale scores were compared across groups (HC, LA‐NC, LA‐VCIND, LA‐VaD) using analyses of covariance (ANCOVA) with age and sex entered as covariates. Bonferroni correction was used to correct for multiple comparisons.

#### Comparison of FA values between LA and HC groups, and among LA groups

The Johns Hopkins University International Consortium of Brain Mapping Diffusion Tensor Imaging 81 atlas used in this study covered the entire skeleton of major white matter fiber tracts.^26^ Mean FA values of all skeleton voxels of the specific tracts were compared between the entire LA sample and the HC participants. A general linear model was used with age and sex entered as covariates and Bonferroni correction performed for multiple comparisons. Separate ANCOVAs for each voxel were also performed to compare the three LA groups with varying levels of cognitive impairments. Age and sex were entered as covariates, and Bonferroni correction was used for post hoc comparisons.

#### Relationship between FA values and cognitive scores in different LA groups

Spearman correlational analysis was conducted between the mean FA values of all skeleton voxels of the specific tracts which have shown a group difference and the MoCA scores in all three LA groups. Mean FA values, age, and sex were entered as independent variables, and MoCA subscale scores were entered as dependent variables. An adjusted *P* value of 0.0071 (0.05/7) was used for each of the seven MoCA subscale scores to correct for multiple comparisons.

## Results

### Participant characteristics

Group characteristics are summarized in Table 1. There were no significant differences across the groups (HC, LA‐NC, LA‐VCIND, LA‐VaD) regarding age, sex and years of education. Significant between‐group differences were observed on CDRS total score (*F = 61, P < 0.01*), MoCA visual‐spatial score (*F = 21.57, P < 0.01*), MoCA naming score (*F = 5.32, P = 0.02*), MoCA attention score (*F = 20.7, P < 0.01)*, MoCA language score (*F = 11, P < 0.01)*, MoCA memory score (*F = 8.38, P < 0.01)*, and MoCA orientation score (*F = 8.7, P < 0.01)*. No significant between‐group difference was found on the MoCA abstraction score (*F = 2.35, P = 0.78)*. Post hoc comparisons revealed significant differences between the HC and LA‐VaD groups on all cognitive scores (*P < 0.02*). No statistically significant difference was found between the HC and LA‐NC groups. Both the CDRS and the MoCA visual‐spatial scores differed among the three LA groups. The LA‐VaD group obtained lower scores than both the LA‐NC group (*P < 0.01, P < 0.01*) and the LA‐VCIND (*P < 0.01, P < 0.01*). The LA‐VCIND group obtained significantly lower scores than the LA‐NC group (*P = 0.022, P = 0.01*) as well as the HC group (*P < 0.01, P < 0.01*). MoCA naming, language, orientation, and attention scores were also significantly lower in the LA‐VaD group compared to the LA‐NC group (*P < 0.01 on all four scores*) and the LA‐VCIND group (*P < 0.02, P < 0.01, P < 0.01, P < 0.01, respectively*). MoCA attention score was also significantly lower in the LA‐VCIND group than the HC group (*P < 0.03*). MoCA memory score did not differ significantly between the LA‐VaD and LA‐VCIND groups, but both groups performed worse when compared with the LA‐NC group (*P < 0.01, P < 0.01*) and the HC group (*P < 0.01, P < 0.01*). MoCA abstraction score was significantly lower in the LA‐VaD group than the LA‐VCIND (*P = 0.02*) and HC (*P < 0.02*) groups (Table 2).

**Table 1:** Demographic and Neuropsychological data for participants.

	HC	LA‐NC	LA‐VCIND	LA‐VaD	ANCOVAs(F/p)
Demographic					
Age	58.8 ± 10.8	58.8 ± 8.2	59.9 ± 11.4	61.2 ± 13.33	0.21(0.89)
Sex(M/F)	19/19	9/9	12/12	9/5	0.31(0.81)
Education	13.1 ± 3.5	12.5 ± 3.2	11.7 ± 2.5	10.7 ± 3.4	2.31 (0.08)
Neuropsychology					
CDR	0.16 ± 0.29	0.25 ± 0.35	0.5 ± 0.001	0.64 ± 0.17	61 (0.001)
Visualspatial	4.16 ± 0.95	4.33 ± 1.03	3.13 ± 1.33	1.71 ± 1.07	21.57 (< 0.01)
Naming	2.92 ± 0.27	2.78 ± 0.73	2.67 ± 0.64	2.21 ± 0.80	5.32 (0.02)
Attention	5.66 ± 0.63	5.39 ± 0.70	5.13 ± 1.12	3.50 ± 1.22	20.7 (< 0.01)
Language	2.21 ± 0.62	2.06 ± 0.73	2.08 ± 0.41	1.07 ± 0.92	11 (< 0.01)
Abstract	1.53 ± 0.65	1.39 ± 0.61	1.54 ± 0.72	1.00 ± 0.78	2.35 (0.78)
Memory	2.95 ± 1.45	3.17 ± 1.34	1.96 ± 1.43	1.21 ± 0.80	8.38 (< 0.01)
Orientation	5.76 ± 0.71	5.83 ± 0.51	5.67 ± 0.64	4.57 ± 1.40	8.7 (< 0.01)

Notes:

HC, Healthy controls; LA, Leukoaraiosis; LA‐NC, cognitively normal; LA‐VCIND, vascular cognitive impairment of none dementia; LA‐VaD, vascular dementia; CDR, Clinical Dementia Rating scale. Data presented as mean ± SD unless noted.

**Table 2:** Demographic and Neuropsychological Post hoc analyses Results.

Neuropsychology	F	Sig**(p)**	Post hoc					
			HC versus NC	HC versus VCIND	HC versus VaD	NC versus VCIND	NC versus VaD	VCIND versus VaD
CDR	61	0.00	0.351	0.00	0.00	0.022	0.00	0.00
Visual‐spatial	21.57	0.00	0.574	0.00	0.00	0.001	0.00	0.00
Naming	5.32	0.02	0.385	0.092	0.00	0.536	0.007	0.021
Attention	20.7	0.00	0.293	0.024	0.00	0.343	0.00	0.00
Language	11	0.00	0.407	0.455	0.00	0.891	0.00	0.00
Abstract	2.35	0.78	0.482	0.931	0.015	0.474	0.112	0.02
Memory	8.38	0.00	0.571	0.006	0.00	0.005	0.00	0.104
Orientation	8.7	0.00	0.761	0.646	0.00	0.508	0.00	0.00

Notes:

HC, Healthy controls; LA, Leukoaraiosis; LA‐NC, cognitively normal; LA‐VCIND, vascular cognitive impairment of none dementia; LA‐VaD, vascular dementia.

### FA values comparison between LA and HC, and among LA sub‐groups

**Figure 1:**
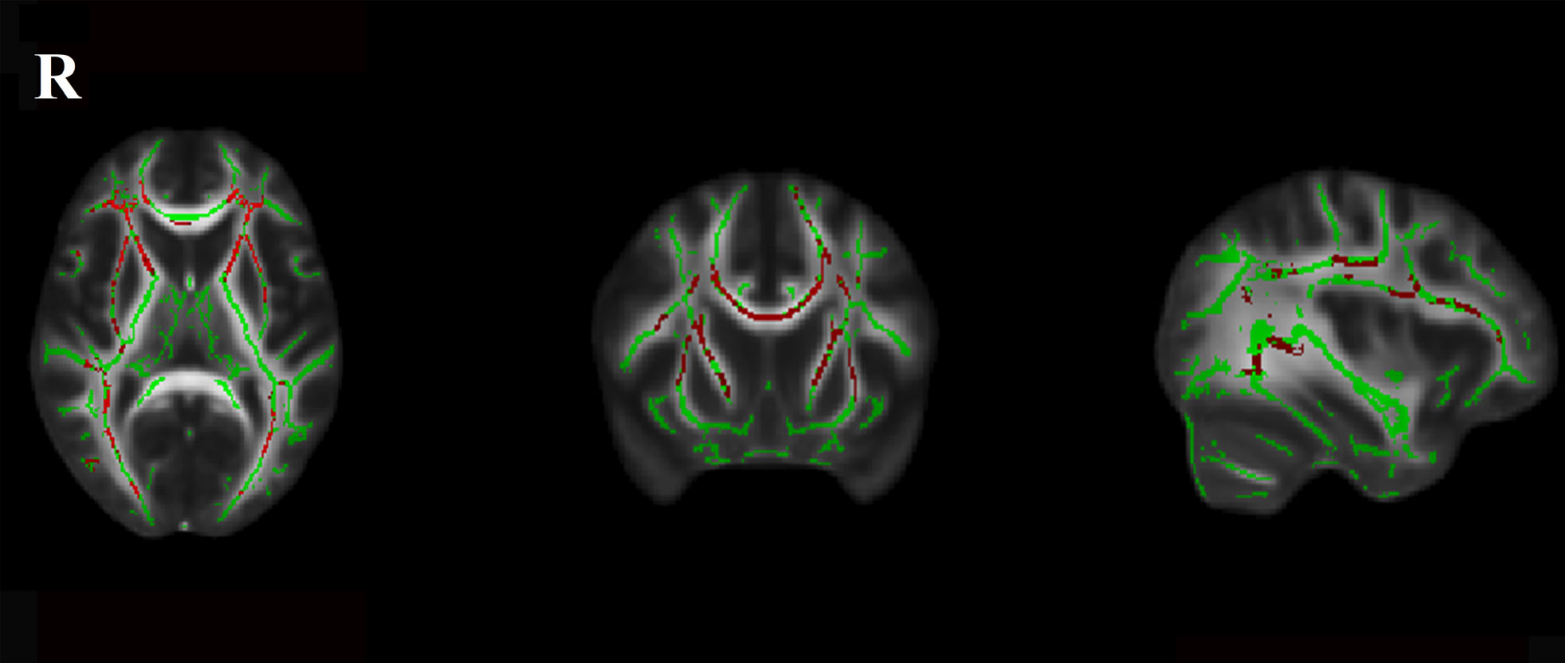
Fractional anisotropy between LA participants and controls. The figure shows white matter fibers’ significant fractional anisotropy reduction (Red Color) in LA participants compared with controls at the TBSS analysis (*P* < 0.05 corrected for multiple comparisons). The white matter tracts are superimposed to the FMRIB58_FA standard provided with FSL.

**Figure 2:**
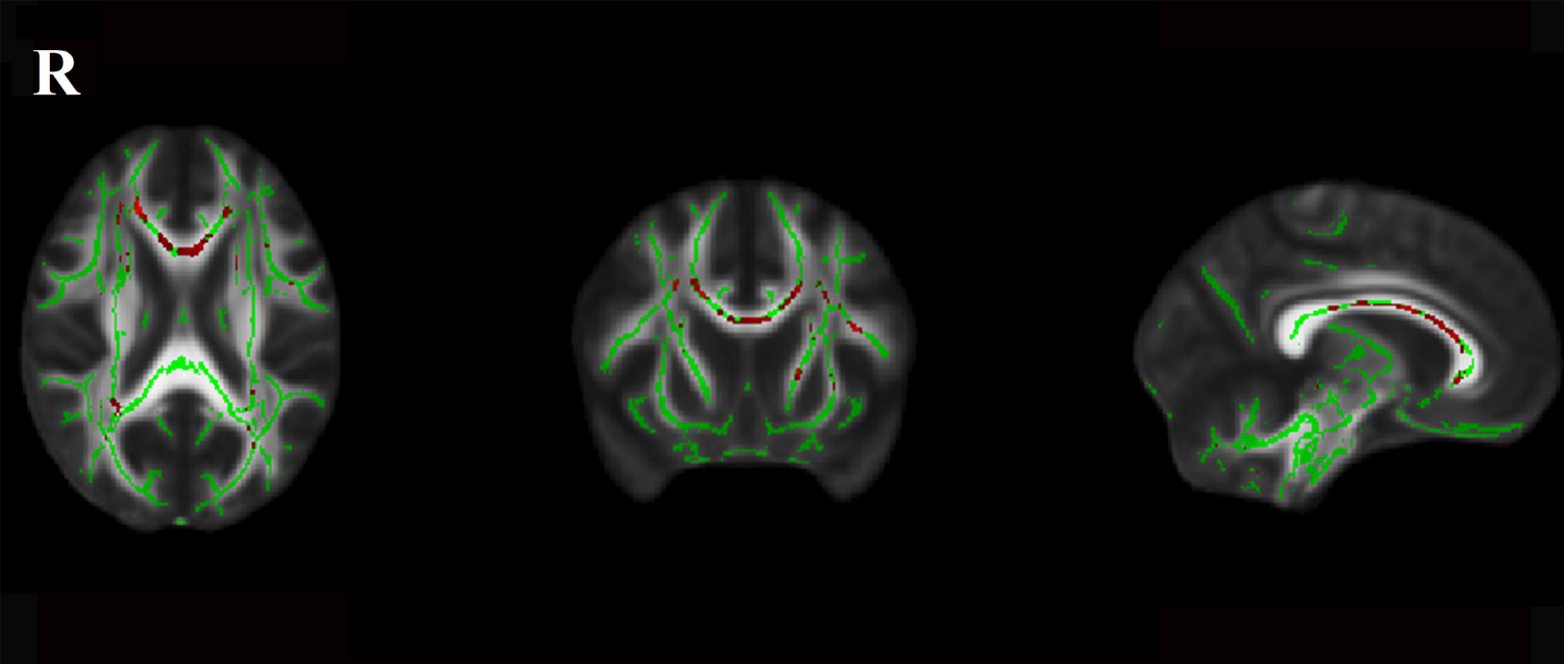
Fractional anisotropy difference among the LA groups. The figure shows white matter fibers’ significant fractional anisotropy reduction (Red Color) among the LA‐NC, LA‐VCIND, and LA‐VaD at the TBSS analysis (*P* < 0.05 corrected for multiple comparisons). The white matter tracts are superimposed to the FMRIB58_FA standard provided with FSL.

**Figure 3:**
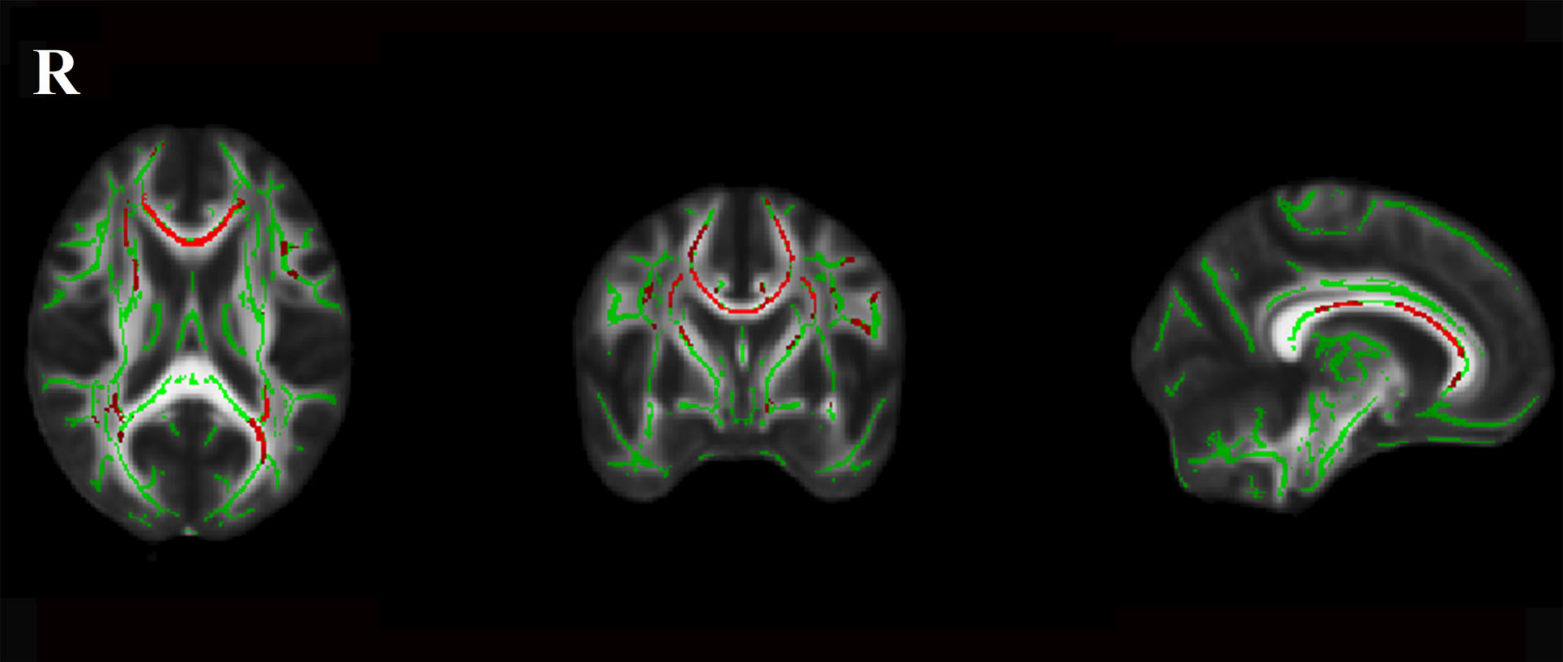
Fractional anisotropy between LA‐NC and LA‐VaD. The figure shows white matter fibers significant fractional anisotropy reduction (Red Color) in LA‐VaD participants compared with LA‐NC at the TBSS analysis (*P* < 0.05 corrected for multiple comparisons). The white matter tracts are superimposed to the FMRIB58_FA standard provided with FSL.

**Figure 4:**
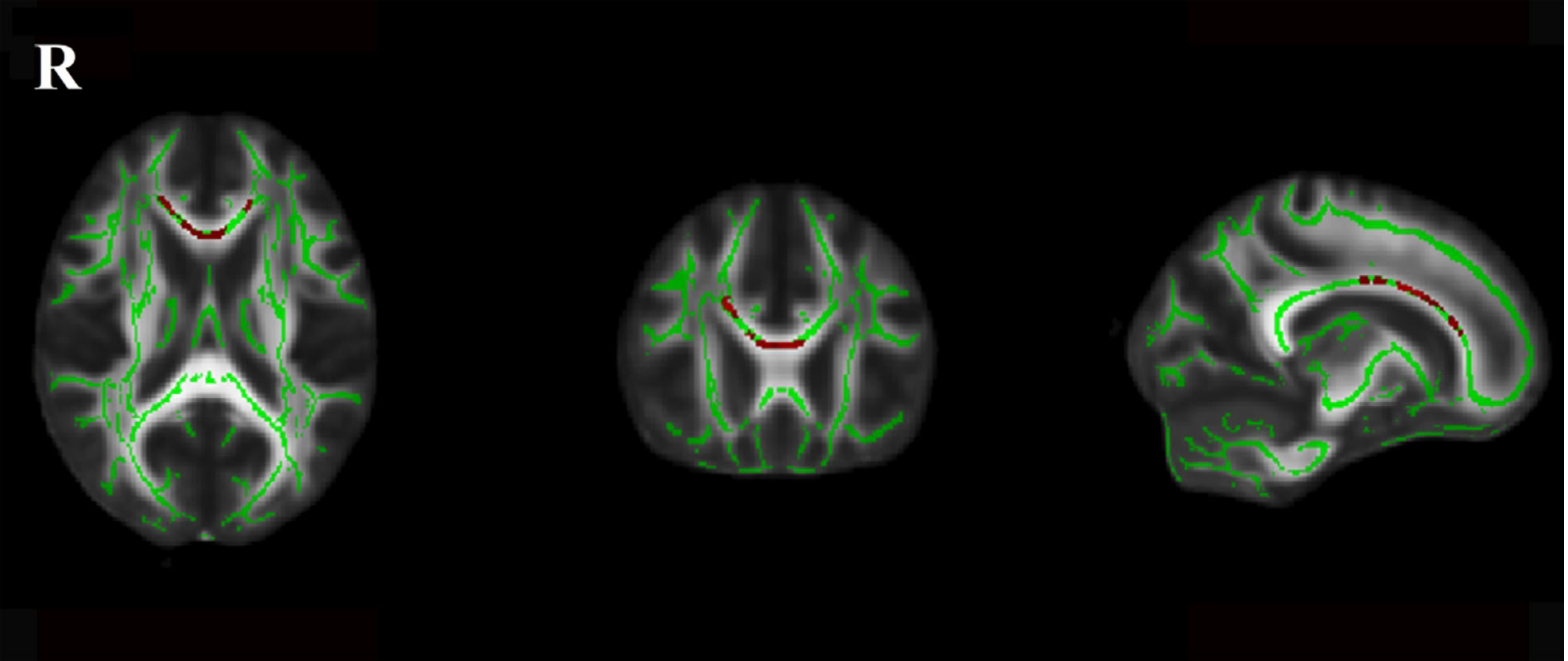
Fractional anisotropy between LA‐VCIND and LA‐VaD. The figure shows white matter fibers significant fractional anisotropy reduction (Red Color) in LA‐VaD participants compared with LA‐VCIND at the TBSS analysis (*P* < 0.05 corrected for multiple comparisons). The white matter tracts are superimposed to the FMRIB58_FA standard provided with FSL.

Significant between‐group differences for the normalized total FA skeleton, regressing out age and sex, were found in the genu, body, and splenium of the CC, bilateral anterior corona radiata, superior corona radiata, posterior corona radiata, anterior limb of internal capsule, external capsule, posterior thalamic radiation, and superior longitudinal fasciculus (Figure 1). Significant differences among the three LA groups were found in the genu of CC (*P < 0.001, F = 12.97*), body of CC (*P < 0.001, F = 13.03*), splenium of CC (*P < 0.001, F = 10.74*), right anterior corona radiata (*P < 0.001, F = 14.75*), left anterior corona radiata (*P < 0.001, F = 9.94*), right superior corona radiata (*P < 0.001, F = 9.41*), left superior corona radiata (*P < 0.001, F = 10.32*), right posterior corona radiata (*P < 0.001, F = 9.35*), left posterior corona radiata (*P < 0.001, F = 7.98*), right anterior limb of internal capsule (*P < 0.001, F = 8.22*), left anterior limb of internal capsule (*P < 0.001, F = 10.98*), right superior longitudinal fasciculus (*P < 0.001, F = 7.89*), left superior longitudinal fasciculus (*P < 0.001, F = 8.03*), right posterior thalamic radiation (*P < 0.001, F = 7.54*), left posterior thalamic radiation (*P < 0.001, F = 9.30*), and left external capsule (*P < 0.001, F = 7.06*) after regressing out age and sex (Figure 2). Post hoc comparisons revealed a reduction in FA values in the LA‐VaD group compared with the LA‐NC group in the same fibers (Figure 3). Significant differences between the LA‐VCIND and LA‐VaD groups were also found in the body and genu of the CC (Figure 4). The FA values did not differ significantly between the LA‐NC and LA‐VCIND groups.

### Relationship between FA values and cognitive scores in different LA sub‐groups

**Figure 5:**
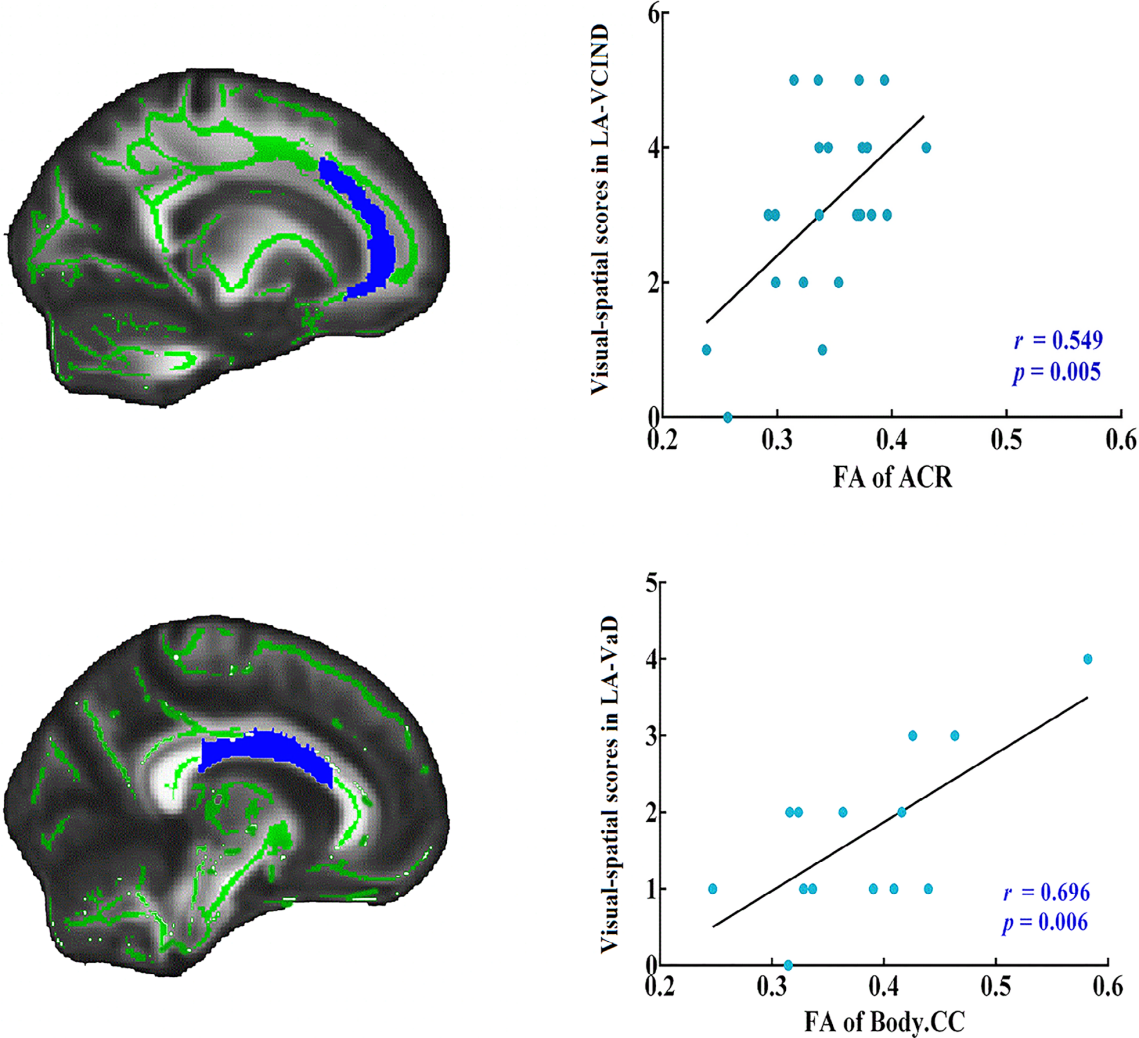
Relationship between FA values and cognitive domain scores in different LA groups. The plots displaying the correlation between areas showing decreased FA values in LA‐VCIND and LA‐VaD and the white matter fibers in the right anterior corona radiata (ACR) and the body of the corpus callosum (CC).

Impairment in the visual‐spatial domain was associated with FA in the right anterior corona radiata in patients with LA‐VCIND (*r* = 0.549, *P* = 0.005), and with FA in the body of the CC in patients with LA‐VaD (*r* = 0.696, *P* = 0.006) (Figure 5). No other significant associations between FA values and MoCA scores were found.

## Discussion

The present study revealed specific regions with abnormal FA values of the white matter fibers in participants with LA compared to the HC. Specifically, the CC (genu, body, and splenium), corona radiata (anterior, superior and posterior), anterior limb of the internal capsule, external capsule, posterior thalamic radiation, and superior longitudinal fasciculus demonstrated significantly greater FA reduction in patients with LA, and these regions maintained the same across LA patients with varying degrees of cognitive impairments. The visual‐spatial performance was correlated with right anterior corona radiata impairment in the LA‐VCIND group. Callosal fiber damage, particularly in the body of the CC, showed notable consistent deficits along with cognitive deterioration in the LA groups and demonstrated regional specificity associated with visual‐spatial impairment. In summary, these findings showed that CC integrity loss in LA, as measured by FA values, held a consistent pattern of specific regional fiber loss across the spectrum of cognitive impairments. The anterior‐posterior (anterior corona radiata to the body of CC) microstructure damage, which progressed along with cognitive degradation, was associated with prominent visual‐spatial impairments, and may be more conducive to characterizing the early changes in dementia with white matter lesion load.

Prior cross‐sectional studies have found white matter alterations in the aforementioned regions when comparing individuals with dementia with healthy individuals.^27^, ^28^, ^29^ The extent of the white matter lesions was found to associate with the severity of dementia and the progression of the disease.^30^, ^31^ Our study demonstrated that using the FA values of the fiber abnormality of those with LA compared to healthy controls, we can also distinguish individuals with LA and the most severe cognitive impairments (i.e., the LA‐VaD group) from those with LA yet have intact cognitive function. Such overlapping abnormalities of fiber bundles were of interest as they reflect changes that maintain stable during the degenerative process of those with LA and develop dementia.

We further found extensive damage in the corona radiata fasciculus (anterior, superior, and posterior) in the participants with LA. The white matter fibers lesion burden and white matter hyperintensity were found to associate with the impairments of fiber‐connected gray matter regions such as the prefrontal cortex in dementia.^5^, ^32^ The corona radiata is part of the limbic–thalamocortical circuitry and contains fibers mainly from the prefrontal cortex regions to basal ganglia and brainstem structures.^33^ Additionally, the posterior thalamic radiation is also part of the thalamocortical projections which connect the thalamic nuclei to basal ganglia and cortical areas such as visual cortical, as well as prefrontal areas.^33^, ^34^ Internal capsule also contains the projection fibers traveling from the thalamus to the cortex. The abnormality in the internal capsule in individuals with early memory impairments may be linked with later cognitive deficits of dementia.^35^ To sum up, fiber damage found in the limbic–thalamocortical circuits likely reflects the pathological neurodegeneration observed in LA‐VaD. We found significant FA reductions in superior longitudinal fasciculus between patients with LA and HC, as well as between LA‐NC and LA‐VaD. Damage to the superior longitudinal fasciculus with abnormal FA indicated disturbance in the frontoparietal regions which may result in cognitive deficits.^36^ A prior study has demonstrated a significant association between the disturbed bilateral superior longitudinal fasciculus integrity and global cognitive decline (Mini‐Mental State Examination and Montreal Cognitive Assessment),^37^ suggesting that the superior longitudinal fasciculus FA may be used as a marker to gauge the severity of cognitive impairments in individuals with LA. The external capsule contains projection fibers connecting prefrontal and temporal areas to basal ganglia.^38^ The decreased anisotropy of individuals with LA suggested that forms of cognitive dysfunction may result from the impairments of the highly interconnected neural circuits encompassing the remote cortices.^37^ Although the results of prior studies have found the difference between individuals with dementia and those who are cognitive normal in external capsule,^28^, ^39^ no significant difference was found between the LA‐NC and LA‐VCIND groups in our study. Accordingly, fiber damage in the external capsule of those with LA may depend more on the severity of cognitive impairments.

It has been suggested that the CC is particularly susceptible to impairments in MCI and AD; however, the relationship between the fiber and topography damage of the CC and the presence and/or severity of cognitive deficits is still controversial.^40^, ^41^, ^42^ Prior studies showed that callosal atrophy is most significant in the splenium of the CC in individuals with AD compared to the HC,^12^, ^41^, ^43^ as well as in the genu and the body of the CC.^40^ Studies on the white matter microstructure also demonstrated decreasing FA values in the CC in individuals with AD and MCI compared to the HC.^29^ In addition, the callosal abnormality was found to be an important predictor of global cognitive impairments in participants with LA.^15^, ^44^ Differential rates of tissue loss in the CC have been observed in individuals with LA and dementia and those with LA but not dementia.^16^ The present study confirmed the loss of integrity in the CC fibers in the LA sub‐groups (i.e., LA‐NC, LA‐VCIND, and LA‐VaD). In addition, the present study extended this line of work by demonstrating that the genu and body of the CC show consistent regional specificity microstructure deficits that correlate with cognitive statuses among individuals with LA.

Loss of white matter microstructure integrity has been observed in our study. Specifically, greater white matter microstructure integrity loss along the anterior‐posterior structure (ACR to the body of the CC) was accompanied by greater cognitive degradation from LA‐VCIND to LA‐VaD and was specifically associated with visual‐spatial impairments. The anterior corona radiata has been recognized as a major white matter fiber connecting the prefrontal lobe to deep gray nuclei and constitutes the frontal‐subcortical circuits. The top‐down visual processing is primarily mediated by the frontal‐striatal circuits.^4^, ^45^ A prior study also highlighted that WMH is more prominent for individuals with MCI than the HC, particularly in the corona radiata.^46^ In addition, loss of white matter tissue composition and integrity has been associated with greater anterior white matter degradation caused by microvascular ischemic events.^40^, ^47^ Thus, the anterior corona radiata fiber damage may correlate with the visual‐spatial impairments early in the cognitive deterioration.

A prior study found that the relationship between anterior CC and cognitive performances is mediated by white matter lesions volume.^17^ Although less significantly, one study found a positive correlation between the degree of cognitive decline assessed by the MMSE and the volume of the anterior body of the CC.^48^ The structural connectivity of the CC is composed of the inter‐hemispheric fibers traversing the subcortical white matter, which mediates the inter‐hemispheric function connectivity.^14^, ^15^ Thus, the participants with LA who also presented with CC abnormality may develop a more severe form of cognitive impairments. CC mediates the dorsal‐ventral pathway of visual‐spatial systems. Longitudinal LA study showed that the decline in the visually mediated processing speed and motor control scores were significantly associated with tissue loss in the mid‐body of the CC.^17^, ^49^ Although a study on Parkinson’s disease indicated that volume loss in the posterior CC segments and potentially altered projections to the parietal‐occipital regions may underlie visuospatial deficits found in dementia,^19^ the present study directly demonstrated the association between the damage of the body of the CC and visual‐spatial deficits in the LA‐VaD group. The stable and extensive FA reduction in the body of the CC in the LA‐VaD participants compared to the HC and LA‐VCIND groups suggested that as patients with LA present with more severe cognitive decline, CC damage may be responsible for specific visual‐spatial cognitive impairment and may distinguish LA‐VCIND from LA‐VaD. Taken together, such primary white matter disconnections in the efferent pathways connecting different cortical regions may be an early marker reflecting the white matter degradation and may be associated with cognitive deficits in the prodromal stages of dementia. These disconnections may later lead to interconnection deficits and extend toward the posterior parts.

The MoCA is a brief cognitive screening tool with high sensitivity and specificity for distinguishing vascular cognitive impairment of none dementia from normal cognitive aging and dementia. It can assess different cognitive domains such as attention/working memory, executive functions, higher‐level language abilities, and complex visuospatial processing.^25^ Our result**s** showed group differences across the cognitive domains assessed except for the abstraction. One possible explanation was that the abstraction impairments may signify cortical gray matter loss and may depend on the severity of white matter lesions load.^50^, ^51^

Several potential limitations of our study and directions for future research should be noted. First, the severity of LA is typically rated visually according to the Fazekas scale and our study did not perform such categorizations.^21^ Further studies are needed to determine whether such categorizations are useful in determining the severity and progression of LA. Second, our study was a cross‐sectional study with a small sample. Evaluation of regions of interest using a longitudinal cohort with a larger and more heterogeneous sample is warranted to confirm our hypothesis. Third, inclusion of individuals with AD and white matter loss and individuals with LA and cognitive impairments who were at an early stage of cerebral small vessel disease (e.g., silent lacunar infarcts) and cortical atrophy should also be considered to increase the generalizability of the findings. Fourth, other metrics that may impact cerebrovascular function such as Body Mass Index were not assessed in this study and may interfere with the current findings.^52^ Finally, amyloid and tau accumulations were not assessed in this study. Further studies should investigate whether amyloid and tau accumulations would impact the FA values in individuals with LA and their correlation with cognitive performance.^53^, ^54^

The present study demonstrated notable fiber damages occurring in participants with LA compared with healthy controls. These fiber damages were believed to maintain stable as the individual progressed to dementia during the disease course. Specifically, the CC fiber loss exhibited stability across the spectrum of cognitive function in participants with LA, and may be more conducive to characterizing the early changes in dementia with white matter lesions load. The anterior‐posterior microstructure damage associated with cognitive degradation was found to predict visual‐spatial impairments and may indicate a clinical manifestation of LA‐related cognitive impairments.

## Author Contributions

1) conception and design of the study: ZW, LB, QL, MZ, YZ; 2) acquisition and analysis of data; ZW, QL, SW, CS; 3) drafting a significant portion of the manuscript or figures: ZW, LB, SW, CS.

## Conflicts of Interest

Nothing to report.

## Supporting information


**Supplementary Material S1.** Detailed clinical characteristics of all LA participants, MRI acquisition, imaging analysis, quality control for DTI data, and tract‐based spatial statistics method.

**Table S1.** Normal‐appearing white matter voxel comparison within LA sub‐groups

**Table S2.** A voxel‐wise correlation of FA values and cognitive domain scores in different LA sub‐groups

## References

[1] Inzitari D , Pracucci G , Poggesi A , et al. Changes in white matter as determinant of global functional decline in older independent outpatients: three year follow‐up of LADIS (leukoaraiosis and disability) study cohort. BMJ 2009;339:b2477 10.1136/bmj.b2477. 19581317 PMC2714680

[2] Zhong G , Zhang R , Jiaerken Y , et al. Better Correlation of Cognitive Function to White Matter Integrity than to Blood Supply in Subjects with Leukoaraiosis. Front Aging Neurosci 2017;9:185 10.3389/fnagi.2017.00185. 28659787 PMC5466957

[3] O'Sullivan M . Leukoaraiosis. Pract Neurol 2008;8:26–38. 10.1136/jnnp.2007.139428. 18230707

[4] Possin KL . Visual spatial cognition in neurodegenerative disease. Neurocase 2010;16:466–487. 10.1080/13554791003730600. 20526954 PMC3028935

[5] Taylor ANW , Kambeitz‐Ilankovic L , Gesierich B , et al. Tract‐specific white matter hyperintensities disrupt neural network function in Alzheimer's disease. Alzheimers Dement 2017;13:225–235. 10.1016/j.jalz.2016.06.2358. 27432800 PMC5319922

[6] Lamar M , Dannhauser TM , Walker Z , et al. Memory complaints with and without memory impairment: the impact of leukoaraiosis on cognition. J Int Neuropsychol Soc 2011;17:1104–1112. 10.1017/S1355617711001123. 21923974

[7] Vemuri P , Lesnick TG , Przybelski SA , et al. Development of a cerebrovascular magnetic resonance imaging biomarker for cognitive aging. Ann Neurol 2018;84:705–716. 10.1002/ana.25346. 30264411 PMC6282853

[8] Wang Z , Williams VJ , Stephens KA , et al. The effect of white matter signal abnormalities on default mode network connectivity in mild cognitive impairment. Hum Brain Mapp 2020;41:1237–1248. 10.1002/hbm.24871. 31742814 PMC7267894

[9] Gorelick PB , Scuteri A , Black SE , et al. Vascular contributions to cognitive impairment and dementia: a statement for healthcare professionals from the American heart association/American stroke association. Stroke 2011;42:2672–2713. 10.1161/STR.0b013e3182299496. 21778438 PMC3778669

[10] Ryberg C , Rostrup E , Sjostrand K , et al. White matter changes contribute to corpus callosum atrophy in the elderly: the LADIS study. AJNR Am J Neuroradiol 2008;29:1498–1504. 10.3174/ajnr.A1169. 18556357 PMC8119069

[11] Lamar M , Catani M , Price CC , et al. The impact of region‐specific leukoaraiosis on working memory deficits in dementia. Neuropsychologia 2008;46:2597–2601. 10.1016/j.neuropsychologia.2008.04.007. 18501390

[12] Lee DY , Fletcher E , Martinez O , et al. Regional pattern of white matter microstructural changes in normal aging, MCI, and AD. Neurology 2009;73:1722–1728. 10.1212/WNL.0b013e3181c33afb 19846830 PMC2788808

[13] Doron KW , Gazzaniga MS . Neuroimaging techniques offer new perspectives on callosal transfer and interhemispheric communication. Cortex 2008;44:1023–1029. 10.1016/j.cortex.2008.03.007. 18672233

[14] Wang Z , Zhang M , Sun C , et al. Single mild traumatic brain injury deteriorates progressive inter‐hemispheric functional and structural connectivity. J Neurotrauma 2019 10.1089/neu.2018.6196. 30931824

[15] Yamauchi H , Fukuyama H , Shio H . Corpus callosum atrophy in patients with leukoaraiosis may indicate global cognitive impairment. Stroke 2000;31:1515–1520. 10884446 10.1161/01.str.31.7.1515

[16] Frederiksen KS , Garde E , Skimminge A , et al. Corpus callosum tissue loss and development of motor and global cognitive impairment: the LADIS study. Dement Geriatr Cogn Disord 2011;32:279–286. 10.1159/000334949. 22262017

[17] Jokinen H , Frederiksen KS , Garde E , et al. Callosal tissue loss parallels subtle decline in psychomotor speed. a longitudinal quantitative MRI study. The LADIS Study. Neuropsychologia 2012;50:1650–1655. 10.1016/j.neuropsychologia.2012.03.020. 22497753

[18] Jokinen H , Ryberg C , Kalska H , et al. Corpus callosum atrophy is associated with mental slowing and executive deficits in subjects with age‐related white matter hyperintensities: the LADIS Study. J Neurol Neurosurg Psychiatry 2007;78:491–496. 10.1136/jnnp.2006.096792. 17028118 PMC2117833

[19] Goldman JG , Bledsoe IO , Merkitch D , et al. Corpus callosal atrophy and associations with cognitive impairment in Parkinson disease. Neurology 2017;88:1265–1272. 10.1212/WNL.0000000000003764. 28235816 PMC5373777

[20] Salvadori E , Poggesi A , Valenti R , et al. Operationalizing mild cognitive impairment criteria in small vessel disease: the VMCI‐Tuscany Study. Alzheimers Dement 2016;12:407–418. 10.1016/j.jalz.2015.02.010. 26079418

[21] Pantoni L , Basile AM , Pracucci G , et al. Impact of age‐related cerebral white matter changes on the transition to disability – the LADIS study: rationale, design and methodology. Neuroepidemiology 2005;24:51–62. 10.1159/000081050. 15459510

[22] Lawton MP , Brody EM . Assessment of older people: self‐maintaining and instrumental activities of daily living. Gerontologist 1969;9:179–186. 5349366

[23] Fazekas F , Chawluk JB , Alavi A , et al. MR signal abnormalities at 1.5 T in Alzheimer's dementia and normal aging. AJR Am J Roentgenol 1987;149:351–356. 10.2214/ajr.149.2.351 3496763

[24] Jokinen H , Koikkalainen J , Laakso HM , et al. Global burden of small vessel disease‐related brain changes on MRI predicts cognitive and functional decline. Stroke 2020;51:170–178. 10.1161/STROKEAHA.119.026170. 31699021 PMC6924941

[25] Nasreddine ZS , Phillips NA , Bedirian V , et al. The Montreal Cognitive Assessment, MoCA: a brief screening tool for mild cognitive impairment. J Am Geriatr Soc 2005;53:695–699. 10.1111/j.1532-5415.2005.53221.x. 15817019

[26] Li S , Tian J , Bauer A , et al. Reduced integrity of right lateralized white matter in patients with primary insomnia: a diffusion‐tensor imaging study. Radiology 2016;280:520–528. 10.1148/radiol.2016152038. 27045987

[27] Mayo CD , Mazerolle EL , Ritchie L , et al. Longitudinal changes in microstructural white matter metrics in Alzheimer's disease. Neuroimage Clin 2017;13:330–338. 10.1016/j.nicl.2016.12.012. 28066707 PMC5200876

[28] Sexton CE , Kalu UG , Filippini N , et al. A meta‐analysis of diffusion tensor imaging in mild cognitive impairment and Alzheimer's disease. Neurobiol Aging 2011; 32: 2322 e2325–2318. 10.1016/j.neurobiolaging.2010.05.019 20619504

[29] Douaud G , Jbabdi S , Behrens TE , et al. DTI measures in crossing‐fibre areas: increased diffusion anisotropy reveals early white matter alteration in MCI and mild Alzheimer's disease. NeuroImage 2011;55:880–890. 10.1016/j.neuroimage.2010.12.008. 21182970 PMC7116583

[30] Targosz‐Gajniak M , Siuda J , Ochudlo S , et al. Cerebral white matter lesions in patients with dementia ‐ from MCI to severe Alzheimer's disease. J Neurol Sci 2009;283:79–82. 10.1016/j.jns.2009.02.314. 19268974

[31] Tomimoto H . White matter integrity and cognitive dysfunction: Radiological and neuropsychological correlations. Geriatr Gerontol Int 2015;15 Suppl 1:3–9. 10.1111/ggi.12661. 26671151

[32] Holland CM , Smith EE , Csapo I , et al. Spatial distribution of white‐matter hyperintensities in Alzheimer disease, cerebral amyloid angiopathy, and healthy aging. Stroke 2008;39:1127–1133. 10.1161/STROKEAHA.107.497438. 18292383 PMC2754400

[33] Olivo G , Wiemerslage L , Swenne I , et al. Limbic‐thalamo‐cortical projections and reward‐related circuitry integrity affects eating behavior: A longitudinal DTI study in adolescents with restrictive eating disorders. PLoS One 2017;12:e0172129 10.1371/journal.pone.0172129. 28248991 PMC5332028

[34] Chaddock‐Heyman L , Erickson KI , Voss MW , et al. White matter microstructure is associated with cognitive control in children. Biol Psychol 2013;94:109–115. 10.1016/j.biopsycho.2013.05.008. 23714226 PMC3742734

[35] Thillainadesan S , Wen W , Zhuang L , et al. Changes in mild cognitive impairment and its subtypes as seen on diffusion tensor imaging. Int Psychogeriatr 2012; 24:1483–1493. 10.1017/S1041610212000270. 22452849

[36] Sjobeck M , Haglund M , Englund E . White matter mapping in Alzheimer's disease: a neuropathological study. Neurobiol Aging 2006;27:673–680. 10.1016/j.neurobiolaging.2005.03.007. 15894407

[37] Meng JZ , Guo LW , Cheng H , et al. Correlation between cognitive function and the association fibers in patients with Alzheimer's disease using diffusion tensor imaging. J Clin Neurosci 2012;19:1659–1663. 10.1016/j.jocn.2011.12.031. 23062795

[38] Schmahmann JD , Smith EE , Eichler FS , et al. Cerebral white matter: neuroanatomy, clinical neurology, and neurobehavioral correlates. Ann N Y Acad Sci 2008;1142:266–309. 10.1196/annals.1444.017. 18990132 PMC3753195

[39] Chua TC , Wen W , Slavin MJ , et al. Diffusion tensor imaging in mild cognitive impairment and Alzheimer's disease: a review. Curr Opin Neurol 2008;21: 83–92. 10.1097/WCO.0b013e3282f4594b. 18180656

[40] Radanovic M , Pereira FR , Stella F , et al. White matter abnormalities associated with Alzheimer's disease and mild cognitive impairment: a critical review of MRI studies. Expert Rev Neurother 2013;13:483–493. 10.1586/ern.13.45. 23621306

[41] Wang PJ , Saykin AJ , Flashman LA , et al. Regionally specific atrophy of the corpus callosum in AD, MCI and cognitive complaints. Neurobiol Aging 2006;27:1613–1617. 10.1016/j.neurobiolaging.2005.09.035. 16271806 PMC3482483

[42] Nishioka C , Poh C , Sun SW . Diffusion tensor imaging reveals visual pathway damage in patients with mild cognitive impairment and Alzheimer's disease. J Alzheimers Dis 2015;45:97–107. 10.3233/JAD-141239. 25537012 PMC4500052

[43] Yoshita M , Fletcher E , Harvey D , et al. Extent and distribution of white matter hyperintensities in normal aging, MCI, and AD. Neurology 2006;67:2192–2198. 10.1212/01.wnl.0000249119.95747.1f. 17190943 PMC3776588

[44] Otsuka Y , Yamauchi H , Sawamoto N , et al. Diffuse tract damage in the hemispheric deep white matter may correlate with global cognitive impairment and callosal atrophy in patients with extensive leukoaraiosis. AJNR Am J Neuroradiol 2012;33:726–732. 10.3174/ajnr.A2853. 22210709 PMC8050450

[45] Kastner S , Ungerleider LG . Mechanisms of visual attention in the human cortex. Annu Rev Neurosci 2000;23:315–341. 10.1146/annurev.neuro.23.1.315. 10845067

[46] Fujishima M , Maikusa N , Nakamura K , et al. Mild cognitive impairment, poor episodic memory, and late‐life depression are associated with cerebral cortical thinning and increased white matter hyperintensities. Front Aging Neurosci 2014;6:306 10.3389/fnagi.2014.00306. 25426066 PMC4224123

[47] Vemuri P , Lesnick TG , Knopman DS , et al. Amyloid, Vascular, and Resilience Pathways Associated with Cognitive Aging. Ann Neurol 2019;86:866–877. 10.1002/ana.25600. 31509621 PMC6899909

[48] Chaim TM , Duran FL , Uchida RR , et al. Volumetric reduction of the corpus callosum in Alzheimer's disease in vivo as assessed with voxel‐based morphometry. Psychiatry Res 2007;154:59–68. 10.1016/j.pscychresns.2006.04.003. 17174533

[49] Courtney SM . Attention and cognitive control as emergent properties of information representation in working memory. Cogn Affect Behav Neurosci 2004;4:501–516. 15849893 10.3758/cabn.4.4.501

[50] Au R , Massaro JM , Wolf PA , et al. Association of white matter hyperintensity volume with decreased cognitive functioning: the Framingham Heart Study. Arch Neurol 2006;63:246–250. 10.1001/archneur.63.2.246. 16476813

[51] Price CC , Tanner J , Nguyen PT , et al. Gray and white matter contributions to cognitive frontostriatal deficits in non‐demented Parkinson's Disease. PLoS One 2016;11:e0147332 10.1371/journal.pone.0147332. 26784744 PMC4718544

[52] Emmerzaal TL , Kiliaan AJ , Gustafson DR . 2003–2013: a decade of body mass index, Alzheimer's disease, and dementia. J Alzheimers Dis 2015;43:739–755. 10.3233/JAD-141086. 25147111

[53] Strain JF , Smith RX , Beaumont H , et al. Loss of white matter integrity reflects tau accumulation in Alzheimer disease defined regions. Neurology 2018;91:e313–e318. 10.1212/WNL.0000000000005864. 29959265 PMC6070383

[54] Kantarci K , Schwarz CG , Reid RI , et al. White matter integrity determined with diffusion tensor imaging in older adults without dementia: influence of amyloid load and neurodegeneration. JAMA Neurol 2014;71:1547–1554. 10.1001/jamaneurol.2014.1482. 25347157 PMC4810441

