# Supplementary material for: Corpus callosum integrity loss predicts cognitive impairment in Leukoaraiosis
Source: Ann Clin Transl Neurol. 2020 Oct 29;7(12):2409–20. doi: 10.1002/acn3.51231 (PMC7732249; doi:10.1002/acn3.51231)
Supplement: Supplementary file 1 — Supplementary Material S1. Detailed clinical characteristics of all LA participants, MRI acquisition, imaging analysis, quality control for DTI data, and tract‐based spatial statistics method. [file ACN3-7-2409-s001.docx]

**SUPPLEMENTAL MATERIAL S1**

**Supplemental Methods**

**Participants**

Based on the MRI images and the scores of the MMSE, the participants were divided into four groups: 18 LA participants with normal cognition (LA-NC, 9 females, mean age of 58.8 ± 8.2 years; MMSE scores from 28 to 30), 24 LA participants with VCIND (LA-VCIND, 12 females, mean age of 59.9 ± 11.4 years; MMSE scores from 23 to 27) and 14 LA participants with VaD (LA-VaD, 5 females, mean age of 61.2 ± 13.3 years; MMSE scores smaller than 23). HC group (MMSE scores from 28 to 30).

**MRI Acquisition and Imaging Analysis**

High-resolution T1-weighted images were obtained for each participant with the following T1-weighted 3D magnetization-prepared rapid acquisition gradient-echo sequence (MPRAGE): repetition time (TR) = 2300 ms, echo time (TE) = 3 ms, flip angle = 8°, slice thickness = 1 mm, field of view (FOV) = 256 mm × 256 mm, matrix size = 256 × 256, single-shot, gradient-recalled echo planar imaging (EPI) sequence with 30 slices covering the whole brain: TR = 2000 ms, TE = 30 ms, flip angle = 90°, thickness = 5 mm, FOV = 200 mm × 200 mm, matrix size = 64 × 64. The DTI were acquired by using a 1.5-T Siemens whole-body MRI system a single-shot, spin echo-based and diffusion-weighted echo planar imaging sequence: TR = 11000 ms, TE = 94 ms, flip angle = 90°, thickness = 3 mm, FOV = 256 mm × 256 mm, matrix size = 128 × 128, two averages. DTI scan (b = 1000 s/mm^2^) were acquired with 30 diffusion gradient orientations and the b = 0 repeated two times. T2WI sequence with the following scan parameters: TR = 4500 ms, TE = 84 ms, flip angle = 120°, matrix = 256 × 256, FOV = 220 mm × 220 mm, slice thickness = 5 mm, slice gap = 1 mm, number of slice = 24. T2WI-FLAIR sequence with the following scan parameters: TR = 8000 ms, TE = 94 ms, flip angle = 90°, matrix = 256 × 256, FOV = 220 mm × 220 mm, slice thickness = 5 mm, and slice gap = 1 mm, number of slice = 24.

The preprocessing steps for DTI data including skull stripping with the brain extraction tool (BET) was applied to the b0 image in each subject. Then, eddy current correction was used to correct for distortions and subject motions on the DTI sequences by aligning the diffusion weighted images to the b0 image. Diffusion tensor fitting (DTIFIT) was used to estimate the diffusion tensor and calculate the scalar DTI statistics. Then, diffusion tensor maps were nonlinearly registered to the standard target using affine alignments and were smoothed using a 6-mm full width at half maximum Gaussian kernel ^1^.

**Quality control for DTI data**

Head motion induces bias in DTI scalar measurements ^2^. Quality assurances were conducted on head motions for all subjects and subjects were excluded from further analysis if they were identified as motion outliers (3 standard deviations (SD) greater than their cohorts). The rotation and translation parameters from each DTI acquisition were obtained using FSL’ s linear registration tool FLIRT of each brain volume to the averaged b0 volume ^3^.

**Tract-based spatial statistics**

For DTI analysis, FA was generated using the Tract-Based Spatial Statistics (TBSS) in the FMRIB Software Library ^4^. Image analysis using TBSS included the following steps: i) non-linear alignment of all subjects’ FA images into a common space using the FMRIB nonlinear registration tool; ii) affine- transformation of the aligned images into standard MNI152 1 mm space; iii) averaging of the aligned fractional anisotropy images to create a 4D mean FA image; iv) thinning of the mean fractional anisotropy image to create a mean FA ‘skeleton’ that represents the centers of all white matter tracts common to the group; and v) thresholding of the FA skeleton at fractional anisotropy 0.2 to suppress areas of extremely low mean fractional anisotropy and exclude those with considerable inter-individual variability.

**Supplemental References**

1. Wang Z, Zhang M, Sun C, Wang S, Cao J, Wang KKW, et al. Single mild traumatic brain injury deteriorates progressive inter-hemispheric functional and structural connectivity. *J Neurotrauma*. 2019

2. Ling J, Merideth F, Caprihan A, Pena A, Teshiba T, Mayer AR. Head injury or head motion? Assessment and quantification of motion artifacts in diffusion tensor imaging studies. *Hum Brain Mapp*. 2012;33:50-62

3. Jenkinson M, Bannister P, Brady M, Smith S. Improved optimization for the robust and accurate linear registration and motion correction of brain images. *Neuroimage*. 2002;17:825-841

4. Smith SM, Jenkinson M, Woolrich MW, Beckmann CF, Behrens TE, Johansen-Berg H, et al. Advances in functional and structural mr image analysis and implementation as fsl. *Neuroimage*. 2004;23 Suppl 1:S208-219
