# Supplementary material for: Corpus callosum integrity loss predicts cognitive impairment in Leukoaraiosis
Source: Ann Clin Transl Neurol. 2020 Oct 29;7(12):2409–20. doi: 10.1002/acn3.51231 (PMC7732249; doi:10.1002/acn3.51231)
Supplement: Supplementary file 2 — Table S1. Normal‐appearing white matter voxel comparison within LA sub‐groups [file ACN3-7-2409-s002.docx]

Table S1 Normal-appearing white matter voxel comparison within LA sub-groups

| White matter fibers | VaD vs NC | VaD vs VCIND | VCIND vs NC |
| --- | --- | --- | --- |
| Genu of corpus callosum | 45 | 5 | 0 |
| Body of corpus callosum | 117 | 31 | 0 |
| Splenium of corpus callosum | 7 | 0 | 0 |
| Anterior limb of internal capsule R | 4 | 0 | 0 |
| Anterior limb of internal capsule L | 7 | 0 | 0 |
| Anterior corona radiata R | 28 | 5 | 0 |
| Anterior corona radiata L | 18 | 1 | 0 |
| Superior corona radiata R | 29 | 8 | 0 |
| Superior corona radiata L | 35 | 5 | 0 |
| Posterior corona radiata R | 8 | 1 | 0 |
| Posterior corona radiata L | 8 | 2 | 0 |
| Posterior thalamic radiation R | 2 | 0 | 0 |
| Posterior thalamic radiation L | 0 | 0 | 0 |
| External capsule R | 1 | 0 | 0 |
| External capsule L | 11 | 2 | 0 |
| Superior longitudinal fasciculus R | 6 | 0 | 0 |
| Superior longitudinal fasciculus L | 2 | 0 | 0 |

Note: Each number in the blank represents the number of different voxels.
