# Supplementary material for: Corpus callosum integrity loss predicts cognitive impairment in Leukoaraiosis
Source: Ann Clin Transl Neurol. 2020 Oct 29;7(12):2409–20. doi: 10.1002/acn3.51231 (PMC7732249; doi:10.1002/acn3.51231)
Supplement: Supplementary file 3 — Table S2. A voxel‐wise correlation of FA values and cognitive domain scores in different LA sub‐groups [file ACN3-7-2409-s003.docx]

Table S2 A voxel-wise correlation of FA values and cognitive domain scores in different LA sub-groups

|  | LA-VaD | | | | | | LA-VCIND |
| --- | --- | --- | --- | --- | --- | --- | --- |
|  | Visual-spatial | Naming | Attetion | Lauguage | Abstract | Memory | Visual-spatial |
| Genu of corpus callosum | 55 | 36 | 20 | 20 | 41 | 25 | 0 |
| Body of corpus callosum | 43 | 13 | 5 | 38 | 21 | 6 | 6 |
| Splenium of corpus callosum | 27 | 13 | 8 | 8 | 2 | 0 | 0 |
| Anterior limb of internal capsule R | 55 | 116 | 40 | 17 | 2 | 2 | 2 |
| Anterior limb of internal capsule L | 362 | 195 | 3 | 111 | 19 | 67 | 64 |
| Anterior corona radiata R | 798 | 279 | 5 | 159 | 59 | 43 | 43 |
| Anterior corona radiata L | 87 | 15 | 7 | 80 | 1 | 22 | 22 |
| Superior corona radiata R | 176 | 12 | 4 | 56 | 11 | 26 | 26 |
| Superior corona radiata L | 29 | 47 | 5 | 248 | 7 | 3 | 3 |
| Posterior corona radiata R | 406 | 61 | 17 | 43 | 3 | 16 | 0 |
| Posterior corona radiata L | 88 | 77 | 8 | 303 | 0 | 2 | 2 |
| Posterior thalamic radiation R | 88 | 20 | 33 | 171 | 3 | 0 | 0 |
| Posterior thalamic radiation L | 98 | 66 | 15 | 67 | 3 | 7 | 6 |
| External capsule R | 0 | 346 | 0 | 377 | 62 | 7 | 3 |
| External capsule L | 235 | 274 | 157 | 457 | 1 | 5 | 5 |
| Superior longitudinal fasciculus R | 187 | 181 | 118 | 488 | 13 | 15 | 12 |
| Superior longitudinal fasciculus L | 417 | 82 | 0 | 280 | 11 | 8 | 3 |

Note: Each number in the blank represents the number of related voxels between the fibers and the cognitive domains.
